# The acute effects of pre- and mid-exercise carbohydrate ingestion on the immunoregulatory stress hormone release in experienced endurance athletes—a systematic review

**DOI:** 10.3389/fspor.2024.1264814

**Published:** 2024-01-26

**Authors:** Tabea Christ, Miriam Ringleb, Simon Haunhorst, Lena Fennen, Paul M. Jordan, Heiko Wagner, Christian Puta

**Affiliations:** ^1^ Department of Movement Science, University of Münster, Münster, Germany; ^2^ Department of Sports Medicine and Health Promotion, Friedrich Schiller University Jena, Jena, Germany; ^3^ NeuroPsycho Immunology Research Unit, Department for Molecular and Cellular Sports Medicine, Institute for Cardiovascular Research and Sports Medicine, German Sport University Cologne, Cologne, Germany; ^4^ Center for Interdisciplinary Prevention of Diseases Related to Professional Activities, Jena, Germany; ^5^ Department of Pharmaceutical/Medicinal Chemistry, Institute of Pharmacy, Friedrich Schiller University Jena, Jena, Germany; ^6^ Center for Sepsis Control & Care (CSCC), Jena University Hospital/Friedrich Schiller University Jena, Jena, Germany

**Keywords:** immunonutrition, endurance exercise, carbohydrates, stress hormones, dietary strategy

## Abstract

**Background:**

In times of physical stress, the body orchestrates a multisystemic regulatory response. The hormones epinephrine and norepinephrine play a role in the immediate regulation chain, while cortisol is involved in delayed regulation. The release of those stress hormones in response to exercise has previously been reported to elicit diverse immune reactions.

**Objective:**

The aim of this systematic review was to examine and present the acute effects of immediate pre- and mid-exercise carbohydrate ingestion on cortisol, epinephrine and norepinephrine levels in experienced endurance athletes.

**Methods:**

A systematic literature search was conducted using PubMed, Cochrane Library and Web of Science in accordance with PRISMA guidelines up to February 2023. Randomized controlled trials in English or German language were included if baseline and at least two follow-up measures of blood plasma or serum of chosen stress hormones (cortisol, epinephrine, norepinephrine) were collected in response to prolonged continuous endurance activity. Eligibility furthermore required an acute carbohydrate ingestion of at least 30 g of carbohydrates per hour no more than 30 min before start of the exercise, as well as a placebo-controlled study design.

**Results:**

Eleven studies of moderate to high quality were included in this review. Carbohydrate ingestion of at least 30 g per hour was able to attenuate rises in cortisol concentration in majority of the included studies. Epinephrine levels were considerably lower with ingestion of carbohydrates compared to placebo in all studies. Norepinephrine concentrations were largely unaffected by acute carbohydrate feeding.

**Conclusion:**

Pre- and mid-exercise ingestion of carbohydrates seems an effective dietary strategy to attenuate rises in cortisol and epinephrine levels and, thus, an effective countermeasure for endurance exercise-induced increases in stress hormone levels.

## 1 Introduction

Exercise constitutes a multidimensional stressor for the organism's homeostatic milieu. On a tissue level, it is usually characterized by metabolic perturbations, such as reactive oxygen species formation, transient oxygen and energy shortage and/or lactic acid accumulation, as well as by inflammatory processes to remove cell debris and induce adaptive remodeling (1–4). Reflecting the multidimensional nature of exercise-induced stress, the maintenance and restoration of homeostasis require a complex interplay of multiple physiological systems.

Essentially involved in this process are neuroendocrine pathways such as the sympathetic-adrenal-medullary (SAM-) axis and the hypothalamic-pituitary-adrenal (HPA-) axis that induce systemic and tissue-level adaptations through a rapid rise in epinephrine and norepinephrine and delayed changes in cortisol levels, respectively (5). Specifically, depending on exercise mode, intensity, and duration, these effector hormones increase energy availability and induce transient changes in leukocyte function and frequency (6). The latter includes, for example, the mobilization of leukocytes into the bloodstream in a biphasic fashion that reflects the distinct kinetics of the stress hormone release (7, 8). The immediate phase of regulation starts with the release of epinephrine and norepinephrine during physical activity. Increased shear stress on the endothelium and adrenergic signaling cause mobilization of all leukocyte subpopulations, accompanied by increased levels of cytokines such as interleukin (IL-) 6, IL-10, IL-1ra and tumor necrosis factor (TNF)-*α* (9–11). Within 90 min to three hours post-exercise, cortisol is involved in the delayed sequence of regulation that is characterized by a continuing increase of neutrophil levels (12, 13). At the same time, blood lymphocyte counts fall below baseline, which can be attributed to their egress from the circulation to peripheral tissues (14, 15). Usually, these immune parameters revert to baseline within 24 h after training (16).

Importantly, existing literature provides evidence that the response of these neuroendocrine pathways to exercise is influenced by the athlete's nutritional status. Research on acute ingestion of carbohydrate supplementation for instance was linked to increased plasma glucose levels, an attenuated cortisol and growth hormone response, fewer perturbations in blood immune cell counts, decreased granulocyte and monocyte phagocytosis and a diminished cytokine response (17–21). Research investigating the effects of a carbohydrate-rich diet, established that this was able to sustain mood state as well as physical capacity during training, hence reducing the symptoms of overreaching (22). In another study on overreaching, researchers observed that an acute high intake of carbohydrates as compared to a low-dose acute intake, was better able to reduce the symptoms of overreaching such as performance decrements and mood disturbances (23).

As carbohydrate ingestion during exercise is commonly understood as a nutritional strategy to counteract a rise in stress hormones in response to exercise, it is likewise hypothesized to mitigate the extent of exercise-induced immune dysfunction (24). The presence and absence of carbohydrates in the body are directly correlated with the number and function of various leukocytes due to the alteration in hormonal regulation, such as the increase of certain catecholamines, the adrenocorticotrophic hormone (ACTH) and cortisol (25).

In order to achieve a beneficial effect from carbohydrate supplementation, high doses should be ingested during prolonged strenuous exercise rather than increasing the daily dietary carbohydrate uptake as it would be done in fueling as claimed by authors in the consensus statement on immunonutrition and exercise (21). To limit depression of neutrophil function or exercise-induced leukocytosis, pre-exercise carbohydrate feeding does not seem to be very effective (26). Moreover, carbohydrate feeding mid-exercise has shown to be a far less effective strategy for short and intermediate activities, as well as for intermittent exercises such as football (27) or rowing (28), but also for strongly fatiguing exercise (29) as opposed to prolonged endurance exercise. A pre- and mid-exercise carbohydrate ingestion of at least 30 g per hour (24, 30), or similarly a beverage with at least six percent carbohydrate content (31, 20, 32) has shown to attenuate the rise in circulating stress hormones such as epinephrine and cortisol from prolonged endurance exercise. On the contrary, exercising under glycogen-depleted conditions has been shown to amplify exercise-induced immune alterations, which might, in some cases, be detrimental to training adaptations (33).

This evidence indicates that the neuroendocrine regulation of immunological parameters might be considerably determined by the athlete's nutritional status, which could, for instance, have substantial beneficial implications for many elite athletes and their performance teams. Factors such as subject fitness but also exercise mode and duration, however, have a decisive impact on stress hormone secretion and will thus affect the immunological outcome, leaving uncertainties for implementation (24). Even though the effectiveness of acute carbohydrate feeding on immune impairments concerning cortisol could be substantiated in the literature so far (17, 20, 27, 29), only a smaller share of studies focused on the role of stress hormones like epinephrine and norepinephrine (28). To the authors' knowledge, there is no systematic review that has investigated and summarized the effects of acute carbohydrate ingestion on immunoregulatory stress hormone release in prolonged endurance activity. Therefore, this systematic review aims to analyze the acute effects of an immediately pre- and mid-exercise carbohydrate ingestion on cortisol, epinephrine and norepinephrine in experienced endurance athletes.

## 2 Methods

This review was planned and conducted according to the Preferred Reporting Items for Systematic Reviews and Meta-Analysis (PRISMA) Statement (34). A protocol was prepared but not publicly registered. A PRISMA protocol can be found in the Supplementary Material.

### 2.1 Eligibility criteria

A PICOS (participants, intervention, comparison, study outcome, study design) approach was used to form the eligibility criteria. Inclusion and exclusion were defined as follows.

(1) participants: inclusion of healthy adult (18 years of age and older) trained endurance athletes with a regular commitment to physical training. Inclusion was independent of form of endurance training, with endurance defined as prolonged, continuous and strenuous exercise, lasting at least 30 min; exclusion of competitive or extremely fatiguing performance, when subjects were non-human or untrained and if performance tests took place under non-normal environmental conditions regarding temperature, altitude and air pressure.
(2) intervention: high carbohydrate consumption during or immediately preceding (30 min or less) the exercise, defined as at least 30 g per hour, 0.5 g/kg body weight per hour, or about one liter per hour of a six percent carbohydrate beverage independent of form of carbohydrate consumption; exclusion of studies with lower than the above-mentioned dose, studies that only provided carbohydrate in combination with other nutritional supplements, or those that supplied them more than 30 min before, or only after termination of the exercise.
(3) comparison: placebo or low carb group; exclusion of studies without placebo-control group
(4) outcome: blood plasma or serum measurement of cortisol, epinephrine, or norepinephrine at rest, directly after exercise cessation, and within one to three hours post-exercise; exclusion of studies missing a baseline and/or follow-up measurement within the adequate time corridor and studies investigating hormone levels from salivary samples.
(5) study design: inclusion of randomized controlled trials, controlled clinical trials, and cross-sectional studies; exclusion of reviews, meta-analysis, meeting abstracts and conference proceedings, ideas, editorials, opinions, records with no identifiable abstract, and all work published in other languages than English or German.

### 2.2 Literature search

The literature search was conducted in February 2023, employing the data-bases PubMed, Web of Science, and Cochrane Library without restrictions on publication date. Search terms were determined from literature scoping with the support of methodological and immunological expertise from the research team (SH and CP). The collection of search terms was based on the PICOS terms and covered the fields of endurance training, carbohydrate, glucocorticoid and catecholamines using common synonyms and controlled vocabulary. Review articles were excluded from the search *a priori*. The exact syntaxes for the respective databases can be found in the Supplementary Material. Additionally, to warrant completeness, reference lists of the included studies as well as other relevant literature, were examined through backward search.

### 2.3 Study selection

**Figure 1:**
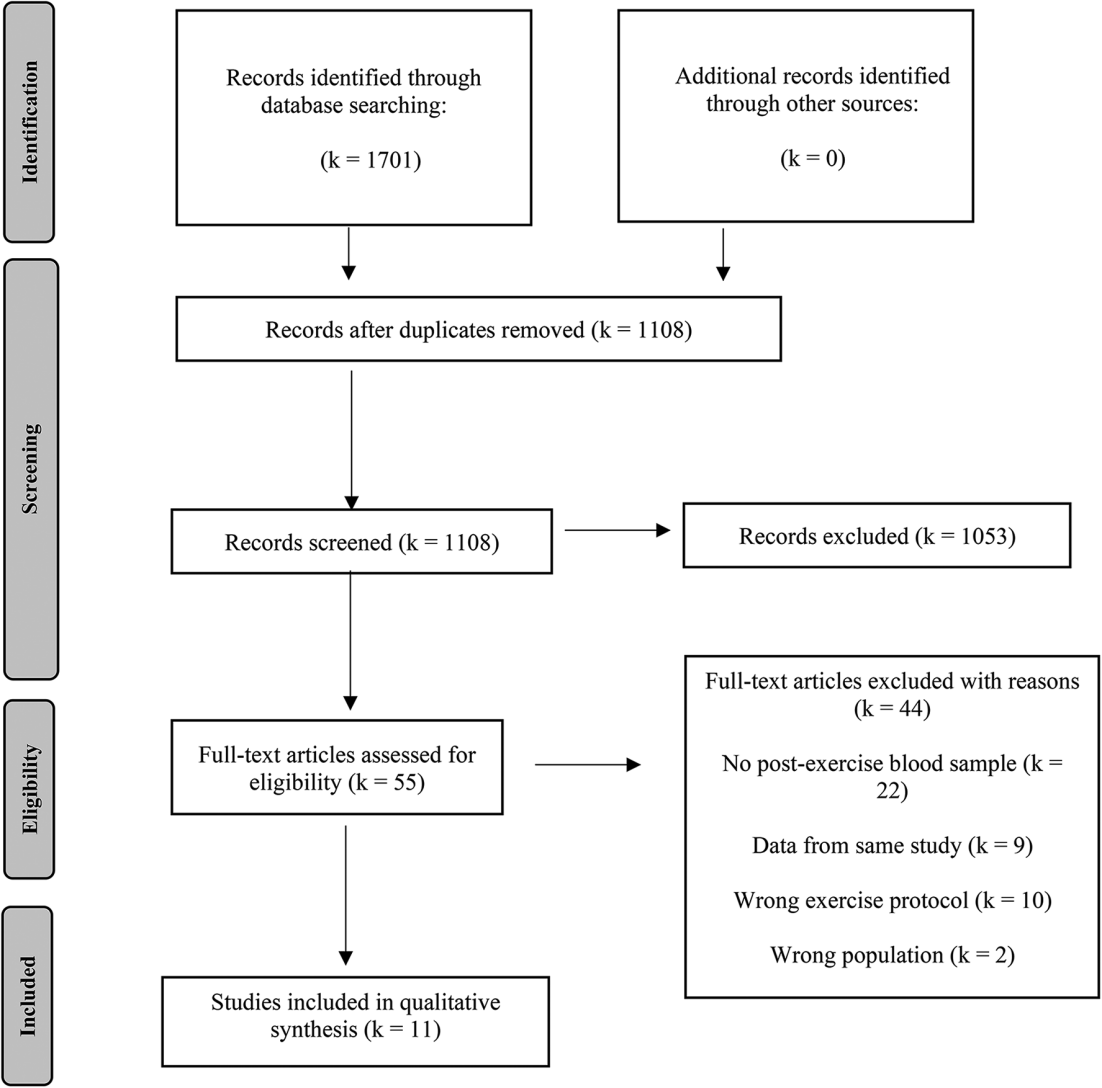
Flow chart of literature search and study selection.

The results of the literature search were uploaded to Rayyan (https://rayyan.qcri.org), a free web-based platform that allows collaborative record management. Within this software, the initial step was to identify and delete duplicates. The selection process was conducted by two reviewers (TC and MR) independently who were blinded to the judgement of the other. In the first phase of study selection, titles and abstracts of all articles were screened. Those studies not eligible for inclusion were excluded. In a second step, full-text screening was conducted for all remaining articles and those that left the need for further inspection. Disagreements were solved by discussions among the team. Those studies considered eligible after full-text examination were included in the review process. Reasons for exclusion in full-text screening are reported in the flow chart (Figure 1).

### 2.4 Data extraction

Data extraction for all included studies was conducted manually in a standardized Microsoft Excel sheet by the first reviewer (TC) and checked for clarity by a second reviewer (MR). The extracted data items include first author, publication year, study design, participant characteristics, exercise protocol, intervention methods, an ingested dose of carbohydrates per hour, the procedure of blood sampling, dietary control, the metabolic stress imposed, investigated hormones and findings for main outcomes from the hormonal markers of interest. If hormone concentrations were not provided in the text or table format, the WebPlotDigitizer digitization program (https://automeris.io/WebPlotDigitizer/) was used to extract plotted data.

### 2.5 Study quality

Risk of bias for each trial was assessed by employing the Physiotherapy Evidence Database (PEDro) rating scale for randomized controlled trials (RCTs). The PEDro scale was used as it is a valid measure of the methodological quality of clinical trials (35). The tool consists of eleven criteria that are scored with either a one (yes), indicating high quality, or a zero (no), indicating low quality of the respective item. The first item regarding specification of eligibility criteria is not used in calculation of the total score, making a score of ten points the optimal one. The risk of bias assessment was conducted by two authors (TC and MR) independently, and disagreements were solved by discussion. Moreover, a grading system was applied to grade the strength of the evidence from RCTs. A score of seven and above resulting from the PEDro indicated a low risk of bias. A score of five or six is considered moderate study quality, while studies scoring below five on the PEDro scale are judged to be of poor methodological quality (36).

### 2.6 Data synthesis

**Figure 2:**
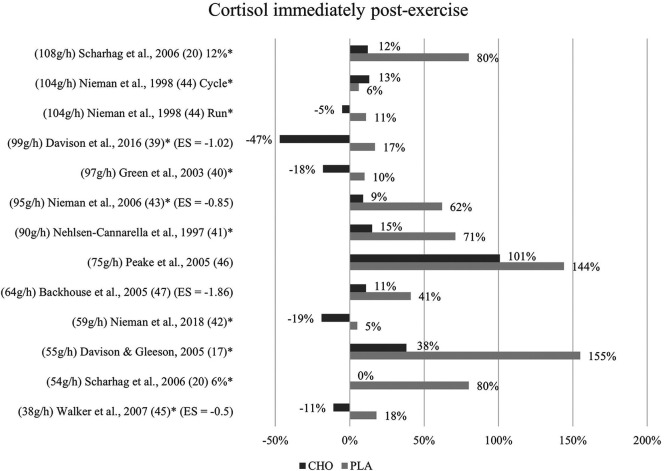
Immediate effects cortisol expressed in change in %; *significant group × time interaction at *p* < 0.05 (differences from baseline values) and Hedge's g effect size.

**Figure 3:**
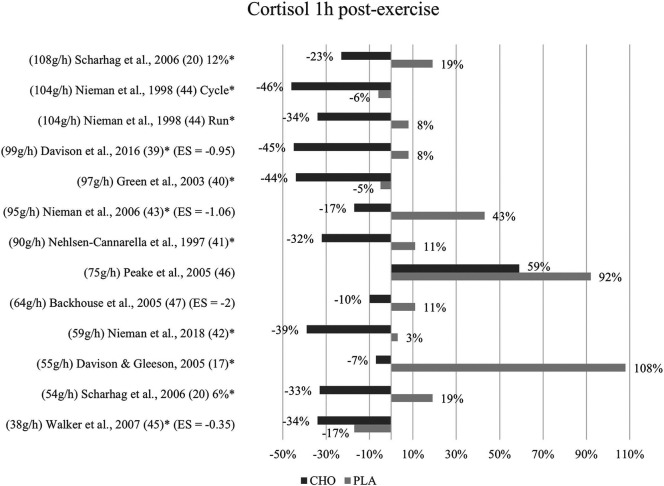
Follow-up effects cortisol expressed in change in %; *significant group × time interaction at *p* < 0.05 (differences from baseline values) and Hedge's g effect size.

**Figure 4:**
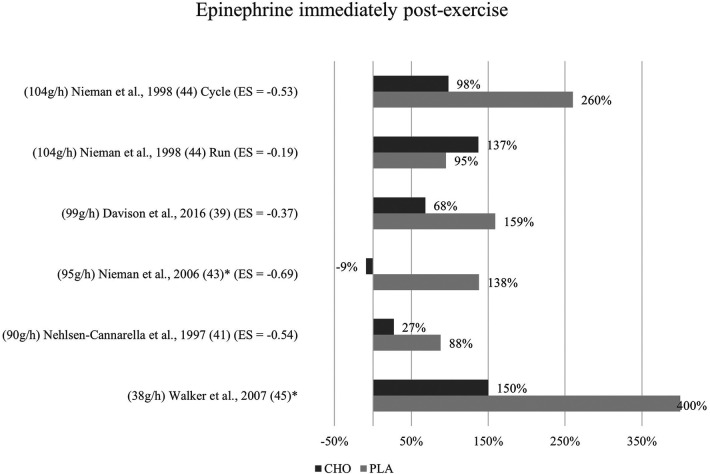
Immediate effects epinephrine expressed in change in %; *significant group × time interaction at *p* < 0.05 (differences from baseline values) and Hedge's g effect size.

**Figure 5:**
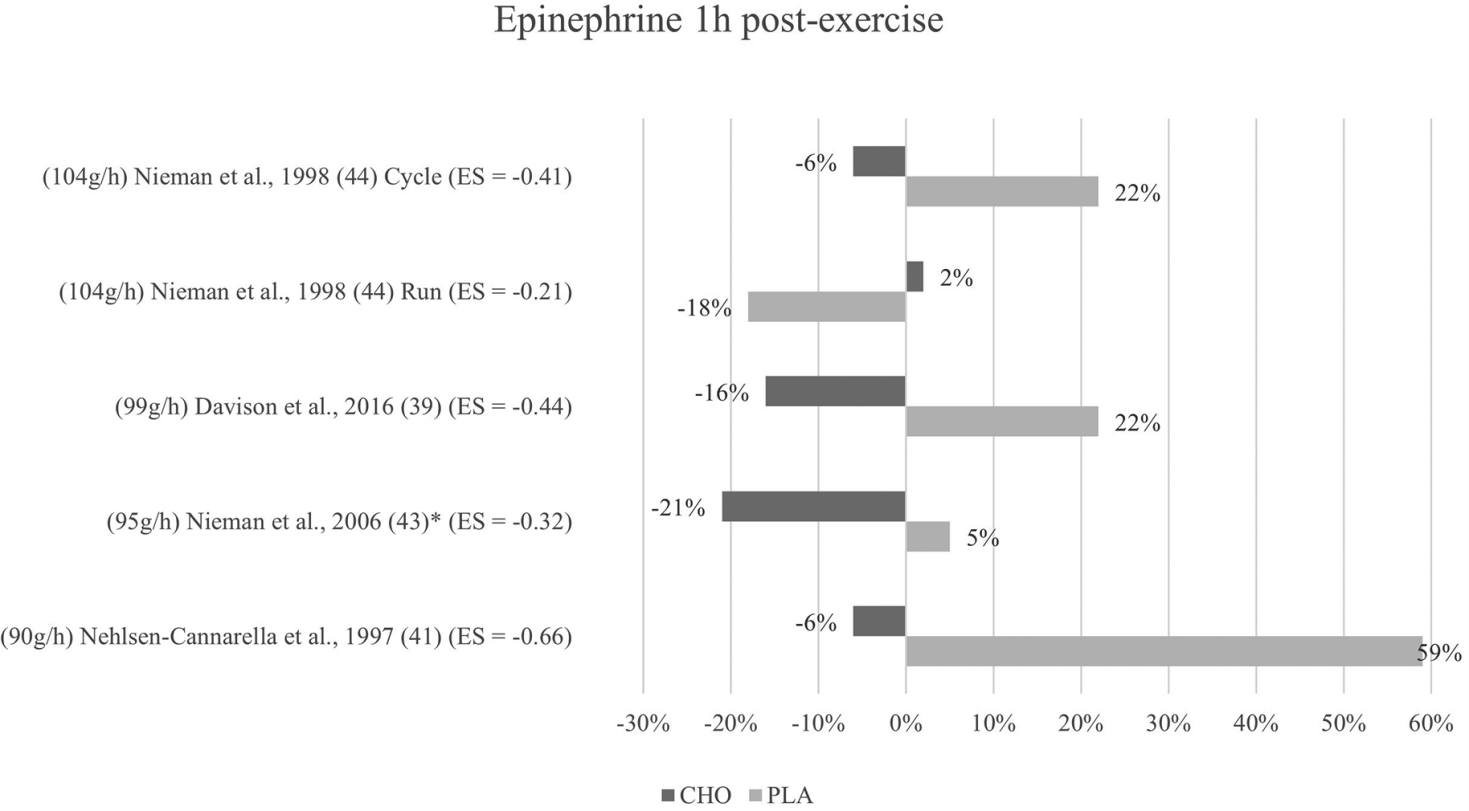
Follow-up effects epinephrine expressed in change in %; *significant group × time interaction at *p* < 0.05 (differences from baseline values) and Hedge's g effect size.

**Figure 6:**
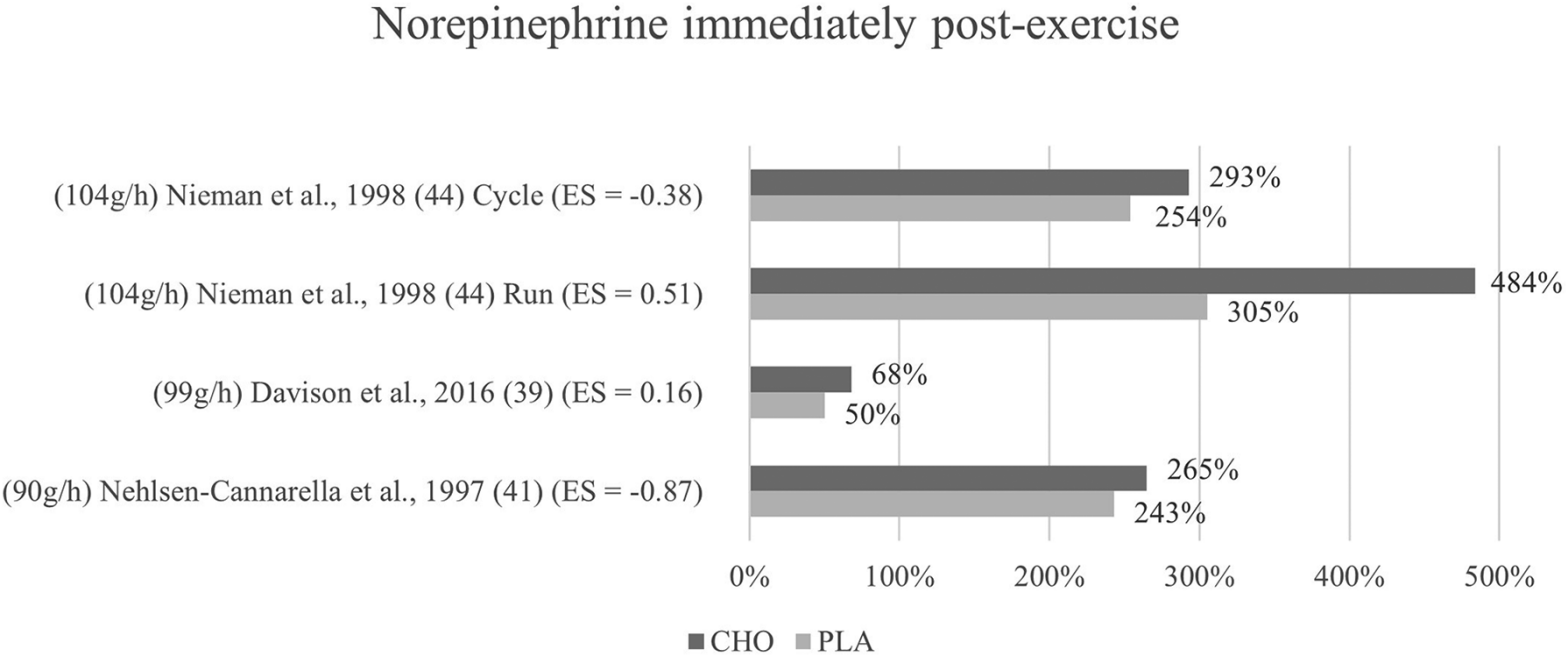
Immediate effects norepinephrine expressed in change in %; *significant group × time interaction at *p* < 0.05 (differences from baseline values) and Hedge's g effect size.

**Figure 7:**
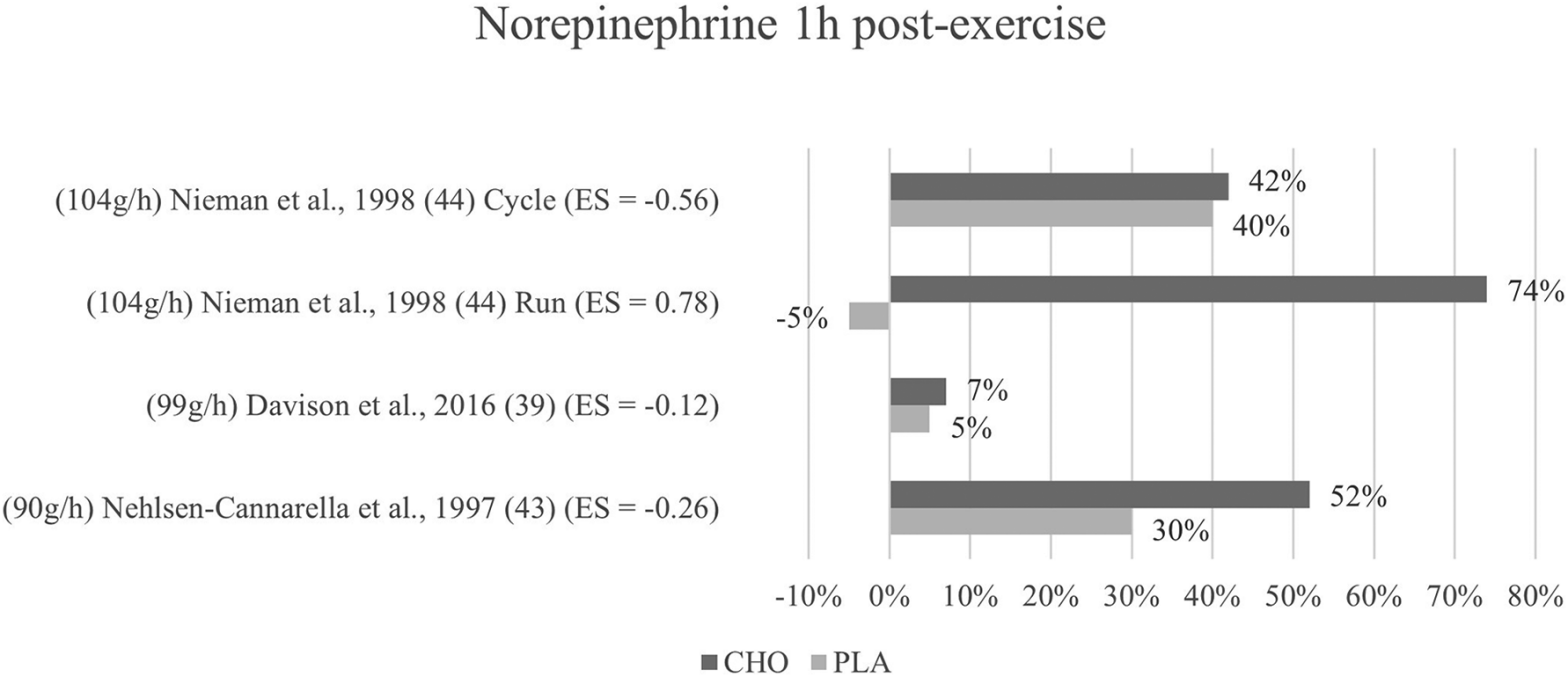
Follow-up effects norepinephrine expressed in change in %; *significant group × time interaction at *p* < 0.05 (differences from baseline values) and Hedge's g effect size.

Data extraction and risk of bias assessment are visually presented in figures and tables. Those results concerning the markers of interest were narratively synthesized. Magnitude and direction of change of blood plasma or serum concentrations of the respective markers were calculated in percentage change and given in extra figures (Figures 2–7). As within the scope of a systematic review statistical calculations are not intended, all ANOVAs mentioned were computed by the individual studies themselves. As none of the included studies reported effect sizes, we calculated Hedge's g, which corrects Cohen's d for small sample sizes for those studies that provided the necessary data mean and standard deviation in tabular form (37). Several studies reported standard error which was converted to standard deviation (38). Effect sizes for the treatment effect between placebo and carbohydrate conditions were calculated for the two post-exercise timepoints respectively.

## 3 Results

### 3.1 Study selection

A total of 1,701 records was identified through literature search (PubMed: 521, Cochrane: 326, Web of Science: 854). Following the removal of duplicates, the titles and abstracts of 1,108 studies were screened. During this process, 1,053 studies were excluded, leaving 55 studies eligible for full-text screening. Further, 44 papers were removed, resulting in eleven articles that met the eligibility criteria to be included in the qualitative and quantitative synthesis (17, 20, 39–47). The search and study selection process are visually depicted in a PRISMA flowchart (Figure 1).

### 3.2 Study characteristics

For each of the eleven included records, summary characteristics are presented in tabulated form (Table 1). The articles were published in English language between the years 1997 and 2018. All studies were placebo-controlled trials, and all but one study (46) explicitly stated randomization of treatment allocation. In six studies (55%) a crossover or counterbalanced design was implemented. Seven studies (64%) explicitly mentioned a double-blind treatment distribution. The total sum of all analyzed subjects from the included studies is 159. Three studies (27%) investigated effects in both men and women, while the remaining eight studies (73%) examined only male subjects. Participants' age ranged from 20 to 45 years (mean age 27 years). All studies specified experience level of the subjects, which ranged from moderately trained and recreationally active to well-trained, competitive athletes. The population within one study was always homogenous.

**Table 1:** Characteristics of included studies.

Reference	Design	N (m/f)	Age (years)M ± SD	Endurance-training status	Exercise protocol	Intervention	CHO (g)/ hour	Control	Blood sampling	Dietary control	Metabolic stress imposed (blood glucose in mmol/l)	Results Cortisol	Results Catecholamines
Backhouse et al. (47)	randomized double-blind counterbalanced	9 (9/0)	25 ± 2	Endurance trained	2 h cycling on electronically braked ergometer at 70% VO2max	CHO (6.4%) solution immediately pre-ex (5 ml/kg bm), every 15 min mid- (2 ml/kg bm), and 5 min post-ex (5 ml/kg bm)	64	PLA ingested at same times/ rates as CHO	blood sample from antecubital vein: at rest, immediately post, 1 h post-ex	subjects overnight fasted, standardized diet before measurements	PLA (pre, immediately post, 1 h post-ex): 5.4, 5.4, 5 CHO (pre, immediately post, 1 h post-ex): 5.2, 6.1, 5.3	no sign group × time interaction; but sign main effect of trial (*p* < 0.05), with CHO trial plasma concentrations lower than PLA trial at both post-ex timepoints	
Davison and Gleeson (17)	randomized single blind crossover	6 (6/0)	25 ± 2	Moderately trained	2.5 h cycling on bicycle ergometer at 60% VO2max	CHO (6%) solution 1 h pre-ex (5 ml/kg bm), immediately pre-ex (2.5 ml/kg bm), every 15 min mid- and immediately post-ex (2.5 ml/kg bm)	55	PLA ingested at same times/ rates as CHO	blood sample from antecubital vein: at rest 1 h pre-ex, immediately pre-ex, immediately post, 1 h post-ex	subjects overnight fasted, standardized diet before measurements	PLA (pre, immediately post, 1 h post-ex): 4.8, 4.1, 4 CHO (pre, immediately post, 1 h post-ex): 4.1, 5.6, 4.6	sign group × time interaction (*p* < 0.01), with CHO group plasma concentrations sign lower than PLA at post-ex (*p* = 0.03) and 1 h post-ex (*p* = 0.02)	
Davison et al. (39)	randomized double-blind	32 (32/0)	28.8 ± 8.1	Recreationally active	2 h treadmill running at 60% of VO2max	CHO (10%) solution 20 min pre-ex (5 ml/kg bm), every 15 min mid- (2 ml/kg bm), and immediately post-ex (5 ml/kg bm)	99	PLA ingested at same times/ rates as CHO	blood sample from antecubital vein: at rest 30 min pre-ex, immediately post, 20 min post, 1 h post-ex	food provided by experimenters 24 h pre-measurement, controlled breakfast 3.5 h pre-ex	PLA (pre, immediately post, 1 h post-ex): 4.5, 4.2, 3.8 CHO (pre, immediately post, 1 h post-ex): 4.2, 5.9, 5.1	sign time and group × time interaction (*p* < 0.001), with CHO group plasma concentrations sign lower than PLA at both post-ex timepoints (*p* < 0.05)	no sign group × time interactions for epinephrine (*p* = 0.857) and norepinephrine (*p* = 0.701), but CHO trials plasma concentrations lower than PLA at both post-ex timepoints for epinephrine only
Green et al. (40)	randomized crossover	6 (6/0)	25 ± 5	Well-trained cyclists	2.5 h cycling on bicycle ergometer at 85% VT (≈ to 67 ± 2% VO2max)	CHO (6%) solution 15 min pre-ex (750 ml), every 15 min mid- (280 ml), and in 1 h recovery post-ex (500 ml)	97	PLA ingested at same times/ rates as CHO	blood sample from antecubital vein: at rest, mid-ex (60 min after start), immediately post, 1 h post-ex	subjects overnight fasted, standardized diet before measurements	PLA (pre, immediately post, 1 h post-ex): 4.5, 3.9, 4 CHO (pre, immediately post, 1 h post-ex): 4.1, 4.9, 6.2	sign group × time interaction (*p* < 0.05), with CHO group plasma concentrations sign lower than PLA at both post-ex timepoints (*p* < 0.05)	
Nehlsen-Cannarella et al. (41)	randomized double-blind	30 (24/6)	41.7 ± 2.6	Experienced marathon runners	2.5 h treadmill running at 75–80% of VO2max	CHO (6%) solution 15 min pre-ex (750 ml), every 15 min mid- (250 ml), in 1.5 h recovery post-ex (500 ml), and in 4.5 h recovery (250 ml)	90	PLA ingested at same times/ rates as CHO	blood sample from antecubital vein: at rest 15 min pre-ex, immediately post, 1.5 h post, 3 h post, 6 h post-ex	subjects overnight fasted, standardized diet before measurements	PLA (pre, immediately post, 1 h post-ex): 4.9, 5.3, 4.4 CHO (pre, immediately post, 1 h post-ex): 4.8, 6.7, 5	sign group × time interaction (*p* = 0.022), with CHO group plasma concentrations sign lower than PLA at post-ex (*p* = 0.073) and 1.5 h post-ex (*p* = 0.054)	no sign group × time interactions for epinephrine (*p* = 0.164) and norepinephrine (*p* = 0.117), but CHO group plasma concentrations lower than PLA at both post-ex timepoints
Nieman et al. (42)	randomized crossover counterbalanced	20 (14/6)	40.4 ± 2.4	Competitive cyclists	75-km cycling time trials (simulated moderately difficult) at 70–75% VO2max	CHO (6%) solution 20 min pre-ex (5 ml/kg bm), every 15 min mid- (3 ml/kg bm)	59	water only ingested at same times/ rates as CHO	blood sample from antecubital vein: at rest 20 min pre-ex, immediately post, 45 min, 1.5 h, 3 h, 4.5 h, 21 h and 45 h post-ex	subjects overnight fasted, standardized diet before measurements	PLA (pre, immediately post, 1 h post-ex): 5.3, 5.2, 4.5 CHO (pre, immediately post, 1 h post-ex): 5.3, 5.8, 4.8	sign group × time interaction (*p* < 0.05), CHO group plasma concentrations sign reduced during first 1.5 h of recovery compared to water	
Nieman et al. (43)	randomized crossover	12 (12/0)	21 ± 1	Trained cyclists	2 h cycling on CompuTrainer Pro bicycle ergometer at 60%–65% Wmax or ∼75% VO2peak	CHO (6%) solution 15–30 min pre-ex (12 ml/kg bm), every 15 min mid- (4 ml/kg bm)	95	PLA ingested at same times/ rates as CHO	blood sample from antecubital vein: at rest 30 min pre-ex, immediately post, 1 h post-ex	standardized diet before measurements, standardized liquid meal on morning of test day (5 h before test)	PLA (pre, immediately post, 1 h post-ex): 5.4, 5.1, 4.9 CHO (pre, immediately post, 1 h post-ex): 5.5, 6.5, 6.2	sign group × time interaction (*p* < 0.001), CHO trial plasma concentrations sign lower than PLA trial at both post-ex timepoints (*p* < 0.05)	no sign group × time interaction for epinephrine (*p* = 0.163), but CHO trials plasma concentrations lower than PLA at both post-ex timepoints
Nieman et al. (44)	randomized double-blind	10 (8/2)	34 ± 2.1	Experienced triathletes	2.5 h treadmill or bicycle ergometer exercise at 75% of VO2max	CHO (6%) solution 15 min pre-ex (12 ml/kg bm), every 15 min mid- (4 ml/kg bm), in 1.5 h recovery post-ex (8 ml/kg bm/h), in 4.5 h recovery (4 ml/ kg bm/h)	104	PLA ingested at same times/ rates as CHO	blood sample from antecubital vein: at rest 15 min pre-ex, immediately post, 1.5 h post, 3 h post, 6 h post-ex	subjects overnight fasted, standardized diet before measurements	PLA (pre, immediately post, 1 h post-ex): 4.9, 4.8, 4.6 (run), 5.2, 4.1, 4.5 (cycle) CHO (pre, immediately post, 1 h post-ex): 4.9, 5.8, 4.8 (run), 4.9, 5.5, 4.6 (cycle)	sign group × time interaction for both modes (p = 0.004), with CHO trial plasma concentrations lower than PLA trial at both post-ex timepoints sign only for cycling (*p* < 0.05)	no sign group × time interactions for epinephrine (*p* = 0.265) and norepinephrine (*p* = 0.232), but CHO group plasma concentrations lower than PLA at both post-ex timepoints for epinephrine only
Peake et al. (46)	double-blind crossover	8 (8/0)	28 ± 4	Well trained runners	1 h treadmill running at 85% of VO2max	CHO (10%) solution immediately pre-ex (250 ml), 20 min mid- exercise (250 ml), 40 min into ex (250 ml)	75	PLA ingested at same times/ rates as CHO	blood sample from antecubital vein: 5 min pre-ex, 5 min post, 1 h post-ex	No info	PLA (pre, immediately post, 1 h post-ex): 5, 6.1, 5 CHO (pre, immediately post, 1 h post-ex): 5.1, 7.5, 4.5	no sign difference in pattern of change btw CHO and PLA, non-sign trend towards higher levels in PLA than CHO immediately post ex	
Scharhag et al. (20)	randomized double-blind	14 (14/0)	25 ± 5	Competitive cyclists	4 h constant-load bicycle trials on outdoor track at 70% IAT (≈ to 61–63% VO2max) (using own bicycles with SRM power meter)	CHO (6 or 12%) solution within 1 h pre-ex (10 ml/kg bm), and every 30 min mid- (5 ml/kg bm)	54 (6% cond); 108 (12% cond)	PLA ingested at same times/ rates as CHO	blood sample from antecubital vein: at rest pre-ex, immediately post, 1 h post, 19 h post-ex	standardized breakfast on all test days 2 h before test	PLA on average between 4.2, 5.2 CHO on average between 4.8, 7.1	sign group × time interaction (*p* < 0.001), with both CHO group plasma concentrations sign lower than PLA at both post-ex timepoints (*p* < 0.05), no sign differences btw 6 and 12% CHO groups	
Walker et al. (45)	randomized double-blind crossover	12 (12/0)	22 ± 1	Recreationally active	2 h cycling on bicycle ergometer at 65% VO2max	CHO (6.6%) solution immediately pre-ex (5 ml/kg bm), at 15, 45, 75, and 105 min mid- exercise (2 ml/kg bm), and 5 ml/kg immediately post-ex	38	PLA ingested at same times/ rates as CHO	blood sample from antecubital vein: at rest pre-ex, immediately post, 1 h post-ex	subjects overnight fasted, standardized food on both days before test	PLA (immediately post, 1 h post-ex): 5.2, 4.8, 4.6 CHO (immediately post, 1 h post-ex): 5, 5.7, 4.5	sign group × time interaction (*p* < 0.05), with CHO group plasma concentrations sign lower than PLA at post-ex (p < 0.05) and 1 h post-ex (*p* < 0.01)	sign group × time interaction for epinephrine (*p* < 0.05) with CHO group plasma concentrations sign lower than PLA

The exercise protocol in all studies was prolonged endurance activity as required for eligibility. Seven studies conducted a cycling (ergometer) intervention, whereas, in three studies, a treadmill running activity was chosen. One study (44) implemented both treadmill running and cycling in their exercise protocol. Exercise duration ranged from one to four hours, with majority of the studies choosing two hours or two and a half hours for exercise duration. In only one study (42) exercise goal was not to complete a pre-determined time but to finish a given distance. Exercise intensity was clearly stated for all included studies and ranged from 60% to 85% of maximum volume of oxygen uptake (VO_2_ max).

All eleven studies provided blood glucose concentration for the same sampling timepoints as the investigated stress hormone markers, which was pre-exercise, immediately post- and one to 1.5 h post-exercise. This allows for an inference about the metabolic stress imposed by the respective exercise protocols. For the carbohydrate group, blood sugar concentration rose notably from pre-exercise to immediately post-exercise and fell back to around baseline levels at one hour post-exercise in all but one study (40), in which glucose increased further after the termination of exercise. In the placebo group, a trend was likewise detectable with seven out of eleven studies observing a gradual decline in blood sugar concentrations from pre-exercise to one hour post-exercise (17, 39, 42–45, 47). In two studies, both of which employed running as the exercise mode, blood glucose concentration increased from pre-exercise to immediately post-exercise and fell back to around (46) or below (41) baseline at one-hour post-exercise. In another two studies employing ergometer cycling as the exercise mode, glucose concentrations were reduced from pre-exercise to immediately post-exercise then climbed back up but stayed below baseline at one-hour post-exercise (40, 44).

For the carbohydrate group, the average blood glucose concentration from all studies immediately after exercise was 6 mmol/L. When separately looking at the two modes of exercise, running, with an average glucose concentration of 6.5 mmol/L seemed to impose considerably higher metabolic stress than cycling with an average concentration of 5.7 mmol/L. At one hour after termination of exercise, averages of the two modes were with 4.8 mmol/L (running) and 5.2 mmol/L (cycling) more drawn together. The average blood glucose concentration from all studies in the placebo group immediately after exercise was 4.8 mmol/L. The separate investigation for the two exercise modes showed that running, with an average glucose concentration of 5.1 mmol/L inflicted higher metabolic stress than cycling with an average concentration of 4.6 mmol/L. At the one-hour follow-up measurement, averages of the two modes were with 4.4 mmol/L (running) and 4.5 mmol/L (cycling) almost identical.

Although not subject to investigation in this paper, a comparison of blood glucose concentration between the placebo and the carbohydrate group revealed that carbohydrate ingestion prior to and during endurance exercise results with an average of 6 mmol/L in considerably higher blood glucose levels than a placebo intake with an average of 4.8 mmol/L.

Throughout all studies, the intervention method was ingestion of a carbohydrate solution pre- and/ or mid-exercise as well as a placebo control ingestion administered at the same time and rate as the experimental treatment. All doses were administered in beverage form. Concentration of carbohydrates ranged from six to 12 percent (mean seven percent), with percent indicating the amount of carbohydrates per liter of beverage (six percent equals 60 g of carbohydrates per liter). To adapt treatment prescriptions to the individuals' body weights, the doses were given in relation to body weight in eight of the eleven studies. Using each study's subject's mean body mass (if provided and used in dose allocation) the administered volumes of fluid ranged from 750 ml (46) to 4,352 ml (44), with a mean beverage volume of 2,715 ml. Taking the different percentage ratios of carbohydrate to fluid into account, we calculated total grams of carbohydrates ingested during the critical blood sampling period. Total amounts ranged from 75 g (46) to 432 g (12% condition) (20), with a mean value of 197 g. Finally, regarding the diverse exercise durations, carbohydrates in grams ingested per hour were calculated to allow the most reliable and precise comparison between the studies. Carbohydrate supplementation in grams per hour ranged from 38 g (45) to 108 g (12% condition) (20), mean grams of carbohydrates ingested per hour was 78 g.

In four studies, the carbohydrate beverage was ingested immediately or five minutes pre-exercise; in six studies within 15–20 min before beginning the exercise. In one case, this was done continuously over a time span of one hour before the start (20). In all of the included studies, additional doses were supplemented during the exercise, mostly every 15 min (73% of the studies); in one study, every 20 min; in another study, every 30 min and in yet another study, further doses were administered at irregular yet predetermined time intervals. All but one study supplied the largest volume of beverage in the initial ingestion, and about half to a third of the initial amount of drink for all further intakes. Although this was not a requirement for eligibility in this review, seven of the included studies supplied further portions of carbohydrate beverages after termination of exercise in the recovery phase.

In terms of blood samples collection, all studies acquired their blood samples using an indwelling venous cannula from the antecubital vein. All studies took at least one resting blood sample. Eight of the eleven studies took additional blood samples immediately and one-hour post-exercise (17, 20, 39, 40, 43, 45–47). Three studies (41, 42, 44) collected blood immediately post-exercise and 90 min post-exercise and, moreover, at increasing intervals up to 45 h after termination of exercise, which are, however, not regarded in this review (42).

The studies' individual methods of analyzing stress hormones from the blood samples differed only slightly. For the analysis of cortisol, five studies used the radioimmunoassay (RIA) technique (40, 41, 43, 44, 47). Another four studies used enzyme-linked immunosorbent assay (ELISA) kits (17, 39, 45, 46). One study determined cortisol by chemoluminescence (20) and one study did not explicitly state the analyzing method (42). In determining the catecholamine concentrations, four studies went by high-pressure liquid chromatography (HPLC) with electrochemical detection (41, 44–46). CatCombi ELISA was used by only one study (39). Another study employed competitive enzyme immunoassay to establish catecholamine concentration (43).Concerning the methodological approach, some studies provided information on dietary control of the subjects. In seven studies (64%), subjects were measured in an overnight fasted state, while three studies (27%) provided a standardized breakfast to the participants before the measurement, the remaining one gave no information on food intake (46). Nine studies (82%) stated that participants adhered to a standardized diet for the days preceding the measurements, and three studies (27%) explicitly asked subjects to refrain from caffeine and alcohol intake before the measurement days. Additionally, nine studies (82%) confirmed that participants avoided strenuous activity at least 24 h before the measurements.

The eligible stress hormones analyzed in this review were cortisol, epinephrine, and norepinephrine. All eleven studies incorporated the analysis of cortisol in their investigations. Epinephrine was analyzed in five studies (45%) and norepinephrine was examined in three studies (27%).

### 3.3 Quality assessment

All included studies clearly defined their research aims. Participant characteristics and employed research methods were defined sufficiently, recruitment strategy and eligibility criteria for participants were, however, only sparsely described in some cases (45–47). Repeatability in terms of nutritional control was warranted in ten out of eleven experiments. Concerning exercise load on the pre-measurement days, study replicability can be assured in nine out of eleven studies. Overall, the employed methodology was able to answer the research question adequately. It is important to mention that stress hormones in response to carbohydrate supplementation were never the only investigated item. Therefore, if no meaningful or significant results regarding stress hormones were discovered, the study often discussed this field only sparsely and instead focused on their other discoveries. All studies made an effort to clearly interpret their findings; explicit comments on clinical significance, however, were made only twice. None of the studies were able to score in point measure and variability as the reporting of effect sizes and confidence intervals was omitted. Scores for the assessment of methodological quality of all included studies are presented in Table 2. All studies were of moderate or high quality according to the PEDro scale, with scores ranging from five to ten out of the awardable ten points; the mean score was 7.4.

**Table 2:** Methodological quality of included studies. 0–5/10 poor quality, 5–6/10 moderate quality, 7–10/10 high quality.

Reference	1. Eligibility criteria specified	2. Random group allocation	3. Concealed allocation	4. Comparability of base	5. Blinding of subjects	6. Blinding of therapists	7. Blinding of assessors	8. Adequate follow-up	9. Intention to treat	10. Between-group analysis	11. Point measure and variability	Total	Study quality
Backhouse et al. (47)	0	1	1	1	1	1	1	1	1	1	0	9/10	High
Davison and Gleeson (17)	0	1	1	1	1	0	0	1	1	1	0	7/10	High
Davison et al. (39)	1	1	0	1	1	1	1	1	1	1	0	8/10	High
Green et al. (40)	1	1	0	1	0	0	0	1	1	1	0	5/10	Moderate
Nehlsen-Cannarella et al. (41)	1	1	0	1	1	1	1	1	1	1	0	8/10	High
Nieman et al. (42)	1	1	0	1	0	0	0	1	1	1	0	5/10	Moderate
Nieman et al. (43)	0	1	0	1	0	0	0	1	1	1	0	5/10	Moderate
Nieman et al. (44)	1	1	1	1	1	1	1	1	1	1	0	9/10	High
Peake et al. (46)	0	0	0	1	1	1	1	1	1	1	0	7/10	High
Scharhag et al. (20)	1	1	1	1	1	1	1	1	1	1	0	9/10	High
Walker et al. (45)	0	1	1	1	1	1	1	1	1	1	0	9/10	High

### 3.4 Cortisol level

#### 3.4.1 Immediate effects

The effects of a pre- and mid-exercise carbohydrate ingestion on cortisol were investigated in all eleven studies. Significant group × time interactions from the individual studies' repeated measures ANOVA, indicating a lower level of cortisol immediately after exercise in the carbohydrate group as compared to the placebo group were documented in nine studies (17, 20, 39–45).

In Backhouse and colleagues (47), a significant main effect for trial was detected, with cortisol concentrations being 28% lower in the carbohydrate group than in the placebo group immediately after exercise. This difference was, however, non-significant in terms of an interaction effect. Moreover, no significant group × time interaction was discovered in Peake et al. (46), although post-exercise cortisol levels in the carbohydrate group (793 nM/L) were lower than those of the placebo group (708 nM/L). (Figure 2).

Mean effect size for circulating cortisol concentrations immediately post-exercise was Hedge's g = −1.06, hence a large effect was computed from four studies of the eleven studies investigating cortisol (39, 43, 45, 47). Negative effect sizes suggest larger effects since lower mean values in blood hormone concentration were favorable. The strongest effect for carbohydrate ingestion when comparing the treatment groups was measured by Backhouse and colleagues (47) with a Hedge's g of −1.86. Large effects were also found in Davison et al. (39) with Hedge's g = −1.02 and in Nieman et al. (43) with Hedge's g = −0.85. Medium treatment group effects were computed from data in Walker et al. (45) with Hedge's g = −0.5, notably the study with the smallest amount of ingested carbohydrates per hour by far.

#### 3.4.2 One to three hours post-exercise effects

A group × time interaction for the follow-up measurements conducted within one to three hours after termination of exercise was found to be significant in nine studies (17, 20, 39–45). Although not significant, cortisol levels in the carbohydrate group (477 nM/L) were lower than in the placebo group (641 nM/L) at one-hour post-exercise (47). In Peake et al. (46), cortisol concentration at one-hour follow-up was likewise lower after ingestion of carbohydrates (563 nM/L) than placebo values (625 nM/L).

Cortisol levels decreased to values even below baseline at one-hour post-exercise within the carbohydrate group in all of the included studies except for one (46). In eight studies, this reduction to values lower than baseline was only present for the carbohydrate supplementation group but not the placebo group. In three studies this was also shown for the placebo control subjects (40, 44, 45). Figure 3 depicts the cortisol concentration change in percent from baseline to one-hour post-exercise. For each figure, the studies were ordered by the amount of carbohydrates ingested in grams per hour, from highest to lowest dose.

Mean effect size for circulating cortisol concentrations one to three hours after exercise termination was Hedge's g = −1.09, emphasizing the large treatment group effect. Similarly, to the immediately post-exercise measurements, the strongest effect was found in Backhouse and colleagues (47) with a Hedge's g of −2.00. Moreover, large effects were found in Davison et al. (39) with Hedge's g = −0.95 and in Nieman et al. (43) with Hedge's g = −1.06. Walker et al. (45) found a small to medium effect with Hedge's g = −0.35.

### 3.5 Epinephrine level

#### 3.5.1 Immediate effects

The effects of pre- and mid-exercise carbohydrate ingestion on epinephrine were investigated in five studies (39, 41, 43–45). The repeated measures ANOVA conducted in the respective studies yielded significant group × time interactions in Nieman et al. (43) and Walker et al. (45), pointing towards a lower epinephrine level immediately after exercise in the carbohydrate group as compared to the placebo group.

No significant group × time interactions were found in the remaining three studies (39, 41, 44). As seen in Figure 4, percentage change of hormone concentration from baseline was less pronounced in these three studies with the exception of running mode in Nieman et al. (44).

Mean effect size for blood concentrations of epinephrine immediately post exercise was Hedge's g = −0.46, showing a small to medium treatment effect for the catecholamine computed from four of the five studies investigating epinephrine (39, 41, 43, 44). Three studies (41, 44, 43) showed moderate effect sizes with Hedge's g values between −0.53 and −0.69. Another study (39) found a small effect (g = −0.37). Running mode of a previous study (44) yielded only a very small effect (g = −0.19).

#### 3.5.2 One to three hours post-exercise effects

For the follow-up measure of epinephrine concentration, group × time interaction effects were only significant for Nieman et al. (43). In Davison and colleagues (39), Nehlsen-Cannarella et al. (41), and Nieman et al. (44) (for cycling but not for running), effects were again non-significant in terms of interaction effect. Lower epinephrine concentrations for this timepoint were measured in the carbohydrate groups than in the placebo groups of the respective studies as shown in Figure 5. All three studies, together with Nieman et al. (43), additionally yielded values lower than baseline for the one-hour post-exercise measurement in the carbohydrate group only. Walker et al. (45) forewent the follow-up measurement of epinephrine.

Mean effect size for blood concentrations of epinephrine from the one to three hours post-exercise measurement was Hedge's g = −0.41 which is a small to medium effect, comparable to the immediate measurement. A moderate effect was detected in one study (41) (g = −0.66). Small to medium effects of Hedge's g between −0.44 and −0.32 were reported in three studies (39, 44, 43). Running mode in the study by Nieman and colleagues (44) once more yielded a small effect (g = −0.21).

### 3.6 Norepinephrine level

#### 3.6.1 Immediate effects

The effects of pre- and mid-exercise carbohydrate ingestion on norepinephrine were investigated in three studies (39, 41, 44). Only in one study (41), differences between the carbohydrate and the control group were noticeable (p = 0.232) whereby the post-exercise levels of norepinephrine in the carbohydrate supplementation group were more increased (484%) than those of the placebo group (305%). Norepinephrine concentration in response to exercise with and without the ingestion of a carbohydrate beverage yielded no interaction effect nor between-group difference in any of the three studies. Immediately after exercise, values were higher with the ingestion of carbohydrates than without in the included studies (39, 41, 44) as shown in Figure 6.

Mean effect size for blood norepinephrine concentrations yielded a Hedge's g = −0.15 for the immediate post measurement, implying that there was no treatment effect for carbohydrate ingestion in any of the studies investigating norepinephrine (39, 41, 44). Individually observed, there were however large differences in treatment effects between the studies. One study (41) had a large treatment effect (g = −0.87). Small to medium effects were found for the cycling mode in Nieman et al. (44) g = −0.38, while a medium treatment effect for the placebo group instead of the carbohydrate group was found in the same study for the running mode (g = 0.51). Similarly, a weak placebo group effect was found in Davison et al. (39) (g = 0.16). Even though, the change in hormone concentrations was higher after carbohydrate ingestion in all three studies, medium to large treatment effects for the carbohydrate group were calculated in two studies (41, 44) (cycling mode). This is due to the slightly varying baseline levels between the two groups, resulting in effectively lower hormone levels for the carbohydrate group despite the higher increase induced.

#### 3.6.2 One to three hours post-exercise effects

Regarding follow-up concentrations of norepinephrine, values were non-significantly increased (74%) after exercise with the ingestion of carbohydrates as compared to without (−5%) in Nieman et al. (44) running mode. For Nehlsen-Cannarella et al. (41), norepinephrine levels were increased by 52% in the carbohydrate group and 30% increased in the placebo group, however without a significant interaction. Near to no effects or differences at all were detected by the cycling mode of Nieman et al. (44) and Davison and colleagues (39) for this time point. Figure 7 depicts the epinephrine concentration change in percent from baseline to one-hour post-exercise.

Mean effect size for blood norepinephrine concentrations at one to three hours post exercise termination yielded a Hedge's g = −0.04, showing that no treatment effect for the ingestion of carbohydrates was detected. However, at a glance into individual effects from the studies, the differences are considerable. The Nehlsen-Cannarella study (41) reports only a small treatment effect for this timepoint (g = −0.26), while Nieman (cycling mode) (44) found a moderate effect (g = −0.56). Likewise, the effect size in Davison et al. (39) (g = −0.12) differs from that of the first measurement. The running mode in Nieman (44) continues to yield a medium to large effect (g = 0.78) for the placebo group as opposed to the carbohydrate group.

## 4 Discussion

The aim of this review was to analyze the acute effects of an immediately pre- and mid-exercise carbohydrate ingestion on selected immunoregulatory stress hormones in experienced endurance athletes. The effects are described by the percentage change of the marker's blood concentration from baseline measurement to immediately post-exercise, as well as one to three hours after exercise completion. For studies where data were available, standardized effect sizes for the difference in response magnitude between placebo and carbohydrate ingestion were also calculated. To our knowledge, this is the first systematic review investigating changes in concentration of cortisol, epinephrine, and norepinephrine in response to prolonged endurance activity in combination with carbohydrate supplementation. This research area is of great importance for endurance athletes competing on all levels, as this nutritional strategy is a simple and inexpensive way to facilitate performance.

Overall, eleven studies investigating three different stress hormones were included in this review. Three of the studies were of moderate quality; the remaining eight were of high methodological quality. Notably, the fact that the majority of the included studies investigated outcome measures other than stress hormones might have further reduced the risk of bias due to publication bias. The stress hormone of primary interest was the glucocorticoid cortisol which was examined by all included studies. Nine out of eleven studies observed a significant group (carbohydrate vs. placebo) × time (baseline vs. immediate post-exercise and one-hour post-exercise) interaction effect as computed by a repeated measures ANOVA, illustrating significantly lower stress hormone concentration after exercise with the supplementation compared to without at both time points. Moreover, the large treatment effect for carbohydrate ingestion at both timepoints emphasizes the positive influence acute carbohydrate supplementation has on the expression of the stress hormone cortisol. Therefore, it can be assumed that pre- and mid-exercise carbohydrate ingestion attenuates the rise in blood cortisol levels.

These findings are in line with the vast majority of other research in this field and can be directly explained by the control of blood and tissue glucose levels. The intake of carbohydrates during exercise was found to reduce the trafficking of leukocyte and lymphocyte subsets and limit the reduction in neutrophil function (27, 48, 49). Research shows that a diet low in carbohydrates increases the magnitude of leukocytosis and the rise in neutrophil to lymphocyte ratio, a common marker for exercise stress (50). This exercise-induced mobilization of leukocytes mediated by elevated stress hormones such as cortisol and undesirable occurrence of stress can likely be mitigated through pre- or mid-exercise carbohydrate ingestion (50, 51). Moreover, previous research discovered a negative correlation between carbohydrate ingestion and plasma cortisol levels, with lacking carbohydrate ingestion inducing more pronounced neutrophilia (50). Several studies included in this review observed an increased glucose concentration with a concurrent weakened cortisol concentration following prolonged exercise with carbohydrate supplementation (45, 44, 42, 40, 39). They suggest this occurs owed to the activation of the HPA-axis, resulting in an increased release of ACTH and cortisol (41). Likewise, another review examining pre-exercise carbohydrate ingestion compared with placebo ingestion in prolonged aerobic exercise demonstrated that carbohydrate uptake is associated with significantly higher plasma glucose levels and an attenuated cortisol response as well as the fast return to baseline and for cortisol even below (52). Such a reduction in stress response may enable an athlete to a better training quality and hence performance (24, 30). It may besides this be assumed that acute carbohydrate supplementation could assist in preventing characteristics of overreaching such as performance and mood decrements and thereby improve overall well-being, which is however so far only confirmed by studies investigating carbohydrate fueling diets (22, 23). However, it bears noting that an acute rise in stress hormones is not *per se* to be prevented. In fact, these hormones play an essential role in allocating energetic resources, orchestrating immune surveillance, and adapting the cardiac output to the physical load, thereby ensuring adequate organ perfusion (6). For people suffering from chronic stress, however, the lessening of stress hormone accumulation may perhaps even entail an improvement in overall health.

Especially following prolonged exercise, immunological impacts of stress hormone levels can be observed. A study included in this review by Davison and Gleeson (17) measured an increase in ACTH, cortisol, and IL-6, as well as a 300% increase in the number of circulating leukocytes induced by a 2.5-hour cycling intervention. Intake of a carbohydrate beverage was able to counteract the rise in post-exercise plasma ACTH and cortisol levels. Such findings are congruent with previous ones, establishing that the HPA-axis activation in response to prolonged exercise is dampened through carbohydrate ingestion (17, 53). This reduction of HPA-pathway activation may also be responsible for smaller leukocytosis and neutrophilia with carbohydrate beverages, as their release is stimulated by cortisol (53, 54). The present review did not examine the effect of carbohydrate ingestion on direct immune parameters; however, stress hormones have a close and meaningful link to some aspects of immune function, thus providing the underlying rationale for investigating carbohydrate supplementation as a countermeasure to exercise-induced immune perturbations under conditions when body carbohydrate stores are challenged.

The availability of carbohydrates in the body does not only exert an indirect effect on the immune system through its influence on circulating levels of cortisol and ACTH but also on catecholamines (50). The catecholamines epinephrine and norepinephrine were of further interest in the present review. Five studies measured the change in blood epinephrine concentration. Only two studies found a statistically significant interaction effect (group × time) indicating that pre-exercise carbohydrate ingestion can be linked to decreased stress response in terms of epinephrine (43, 46). In the remaining studies, post-exercise epinephrine concentration was likewise decreased with the consumption of carbohydrate beverages as compared to the control group, however without this interaction being significant. Furthermore, calculated effect sizes signify that small to medium treatment effects for carbohydrate ingestion were found and that these effects were larger at the immediately post-exercise measurement than at one-hour post. From a physiological point of view, the catecholamines exert an immediate effect on exercise-induced biphasic stress regulation by initiating leukocytosis, especially with lymphocytosis and monocytosis (55). It is suggested that the leukocyte redistribution is mediated by the elevated plasma epinephrine level rather than cortisol or growth hormone (51). Correspondingly, cells that are mobilized in response to exercise stress share phenotypical commonalities, such as a high expression level of adrenoceptors and chemokine receptors and high maturation profile (33).

When investigating the second of two 90-minute cycling bouts regarding immunological blood markers, it was observed that the pre- or mid-exercise ingestion of carbohydrates compared to a placebo better attenuated the responses of plasma epinephrine among other stress hormones and thereby lessened the leukocytosis and monocytosis (51). It has frequently been observed for epinephrine concentration to return to baseline in a shorter period of time than norepinephrine, for instance (39, 41, 44).

Lastly, norepinephrine was investigated by three of the eleven included studies in this review. However, contrary to the other stress hormones, no significant effects, indeed not even evident differences between the ingestion groups, were discovered in the included studies. For norepinephrine, it was, in fact, the carbohydrate beverage group that exhibited higher levels of circulating hormone concentration post-exercise at both follow-up time points when compared to the placebo group. This is likewise highlighted by the effect sizes. Overall, no effect for treatment group could be detected for the supplementation of carbohydrates during exercise. Although, measured effect sizes differed among the three studies, it was striking that even large negative effects suggesting a treatment effect for the placebo group were found. However, both catecholamines showed a significant time effect in most of the studies, especially noticeable for the placebo groups, signifying a typical exercise-stress response (56). Literature suggests that catecholamines are strong immunoregulatory hormones, with endurance exercise exerting a direct effect on them (7, 57). Such activity is associated with strong increases in plasma epinephrine and norepinephrine concentration as well as leukocytes and leukocyte subpopulations (58).

The present review shows that the two catecholamines seem to react dissimilarly in response to carbohydrate availability. While the epinephrine rises after carbohydrate intake were attenuated, the norepinephrine levels were intensified for the same ingestion. The phenomenon that epinephrine rises can be reduced by carbohydrate feeding while norepinephrine rises cannot, has been found in the literature before (56, 59, 60). It is, therefore, conceivable that the stress response initiated by the SAM-axis entails a catecholamine-mediated effect that is more strongly expressed or detectable in blood plasma for epinephrine than it is for norepinephrine (51). However, future research in this field is warranted, and the dissimilar effect of carbohydrate consumption in terms of attenuation of one catecholamine and intensification of the other could so far not be explicitly explained by the existing literature.

Nevertheless, it became apparent that carbohydrate ingestion was much more beneficial in lowering cortisol levels than those of catecholamines. Literature has reported cases in which pre-exercise carbohydrate feeding seemed ineffective in limiting the exercise-induced leukocytosis or depression of neutrophil function (26). From a physiological point of view, those events occur in the immediate phase of the biphasic immunological stress regulation in which the catecholamines play a predominant role. It can therefore be assumed that this first chain is less affected by carbohydrate supplementation, whereas the ingestion of carbohydrates exerts a greater weakening effect on the secondary pro-inflammatory cascade leading to diminished cortisol levels (24).

### 4.1 Limitations

A possible limitation of this systematic review is that in some experiments, subjects have fasted overnight, and in others, they received a standardized meal a few hours before the tests. Furthermore, the large range in the amount of carbohydrate ingestion may have impaired the comparability between studies. However, since this review focused rather on comparing placebo and supplementation groups and the pre-post-exercise percentage change within one experiment, it is somehow possible to make accurate interpretations. Especially the bar graphs for cortisol (Figures 2, 3) show that differences between the two ingestion groups were not necessarily the biggest with the highest dose of carbohydrate administered. Moreover, the studies slightly differed in their methodological approach in terms of blood sampling time points but also exercise modalities and thus intensity and duration of the physical effort examined, but again as the conditions for a placebo and carbohydrate group within one investigation were the same, the interpretations are still useful. Another limitation that can be identified is the diverse training status among participants. Even though, subjects within one study were homogenous regarding their training background, larger differences may be found between the groups of subjects from the various studies. Overall, it has to be admitted that to date, carbohydrate ingestion has not been effective in yielding a reduction in upper respiratory tract infections from prolonged activity (24). Finally, a major limitation to this systematic review is the lack of confidence intervals, effect sizes and variances from the individual studies. Unfortunately, the reporting of results within the included studies was not optimal as extensive point measures and variabilities were not provided by any study. This in turn lead to weaknesses in the reporting of results for the review itself as *p*-values together with means were the only indication to synthesize results from. Due to the lack of reported standard deviations in some studies, effect sizes could only be calculated for a fraction of the included studies.

## 5 Conclusion

Physiological stress experienced by an endurance athlete is reflected by the immune system. Prolonged endurance activity leads to noticeable increments in blood plasma concentrations of the stress hormones cortisol, epinephrine, and norepinephrine directly post-exercise as well as some hours after exercise termination. A great body of research has confirmed the hypothesis that an immediate pre- or mid-exercise carbohydrate ingestion attenuates the rise or even lowers the stress hormone level throughout the activity. Due to the intimate link between stress hormones and some aspects of immune function, there is a great basis for this research topic to investigate carbohydrate ingestion as a pre-exercise dietary strategy. Studies in this review indicated that carbohydrate beverage ingestion with a dose of 50–100 g per hour consumed continuously throughout a prolonged exercise program is effective in reducing the activation of hypothalamic pituitary adrenal activation and thereby downregulating the increase in stress hormones and inflammatory cytokines.

Moreover, a reduction in overreaching symptoms commonly comes with a carbohydrate-rich diet enabling the athlete to work harder and longer. All these findings strongly suggest that carbohydrate ingestion can improve performance by enhancing the athlete's training quality as well as overall health, and well-being. Such properties are valuable for athletes preparing for competition while maintaining a robust immune system and avoiding illness. These implications provide an inexpensive and straightforward nutritional method. Majority of research in this domain concludes that the clinical significance of carbohydrate supplementation during exercise and its immunomodulatory effects concerning the hormonal pathway awaits further research. All in all, it must be acknowledged that acute carbohydrate supplementation as a nutritional aid can be considered a partial countermeasure for exercise-induced immune perturbations or immunosuppression.

## Data Availability

Publicly available datasets were analyzed in this study. This data can be found here: Data bases: PubMed, Web of Science, Cochrane Library.
